# Supplementary material for: Ultrastructure of human brain tissue vitrified from autopsy revealed by cryo-ET with cryo-plasma FIB milling
Source: Nat Commun. 2024 Mar 26;15:2660. doi: 10.1038/s41467-024-47066-1 (PMC10965902; doi:10.1038/s41467-024-47066-1)
Supplement: Supplementary file 1 — Supplementary Information [file 41467_2024_47066_MOESM1_ESM.pdf]

# Ultrastructure of human brain tissue vitrified from autopsy revealed by cryo-ET with cryo-plasma FIB milling


## Authors

Benjamin C. Creekmore[1,2,3#], Kathryn Kixmoeller[2,3#], Ben E. Black[2,4], Edward B. Lee[1*], Yi-Wei Chang[2,4*]

## Affiliations

[1]Translational Neuropathology Research Laboratory, Department of Pathology and Laboratory Medicine, Perelman School of Medicine, University of Pennsylvania, PA, USA.

[2]Department of Biochemistry and Biophysics, Perelman School of Medicine, University of Pennsylvania, PA, USA.

[3]Biochemistry and Molecular Biophysics Graduate Group, Perelman School of Medicine, University of Pennsylvania, PA, USA.

[4]Institute of Structural Biology, Perelman School of Medicine, University of Pennsylvania, Philadelphia, PA, USA.

[#]Authors contributed equally

*Correspondence: edward.lee@pennmedicine.upenn.edu (Edward B. Lee), ywc@pennmedicine.upenn.edu (Yi-Wei Chang)

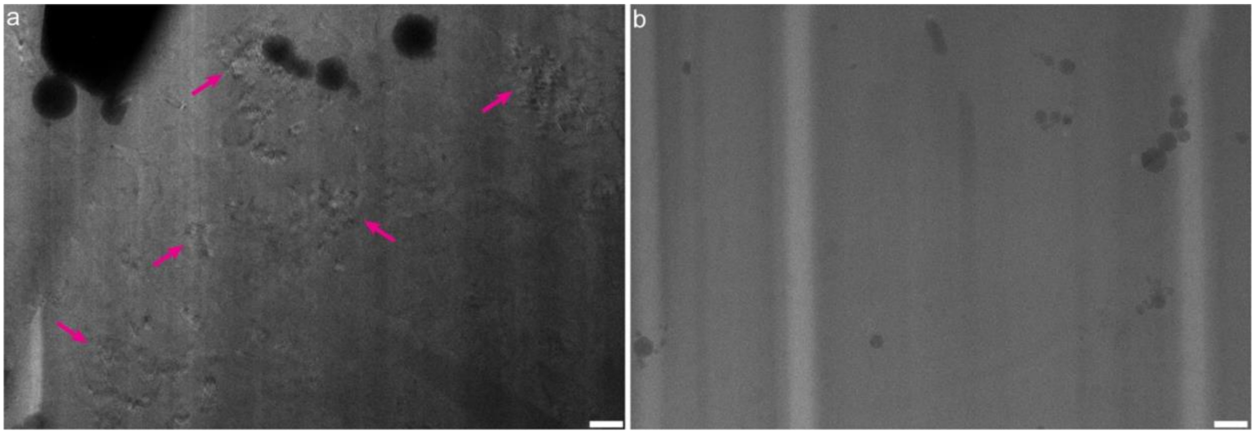


**Supplementary Fig. 1: Lamellae prepared from tissue incubated with 10% glycerol show signs of crystalline ice whereas lamellae prepared from tissue incubated with 20% glycerol and 1M trehalose show no signs of crystalline ice. a** 2D projection image of lamella from tissue frozen after 15 min incubation in 10% glycerol showing Bragg reflections indicative of crystalline ice (pink arrows). **b** 2D projection image of lamella from tissue frozen with incubation of 20% glycerol and 1M trehalose showing no evidence of crystalline ice within the lamella. 17 lamellae across four separate autopsy cases showed no evidence of crystalline ice. Scale bars, 500 nm.

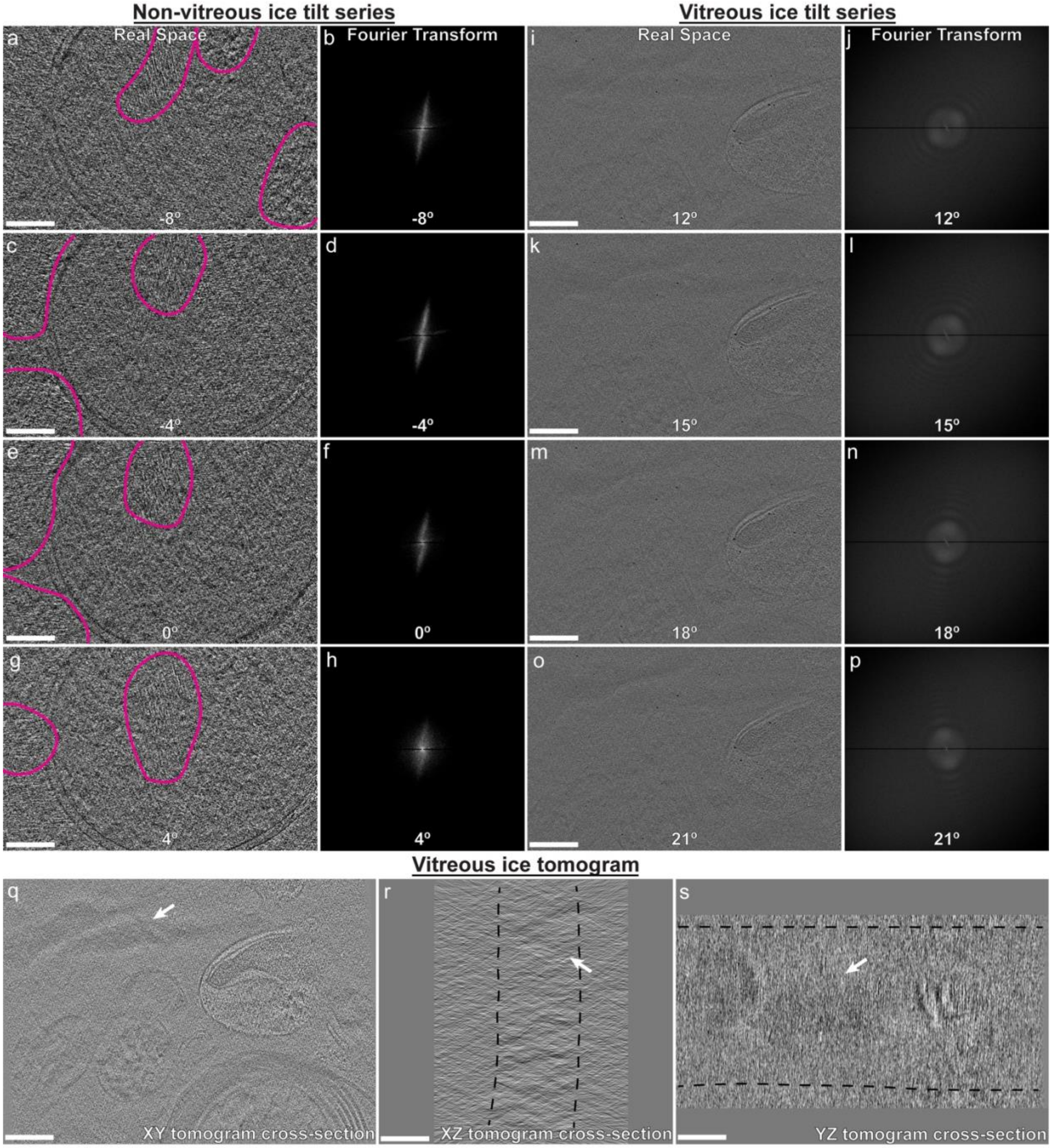


**Supplementary Fig. 2: Lamellae prepared with 20% glycerol and 1M trehalose do not appear vitreous at different tilt angles and in tomographic reconstruction.** Multiple real space and corresponding Fourier transform images from tilt angles from a tilt series taken on a mammalian cell that was not fully vitrified (**a-h**) and on a lamella prepared from tissue incubated with 20% glycerol and 1M trehalose (**i-p**). The mammalian cell tilt series shows Bragg

reflections in real space (**a, c, e, g**; pink outlines) and signs of crystalline ice diffraction in Fourier space (**b, d, f, h**). The tissue lamella (**i-p**) shows no indication of crystalline ice within the lamella. The tilt series shown in **i-p** are shown as a reconstructed tomogram with XY (**q**), XZ (**r**), and YZ (**s**) cross-sections of the same tomogram area. The same electron dense material is noted in each cross-section (white arrow). The surface of the lamella is noted in **r** and **s** by black dashes. 40 reconstructed tomograms showed no evidence of crystalline ice. Scale bars, 200nm.

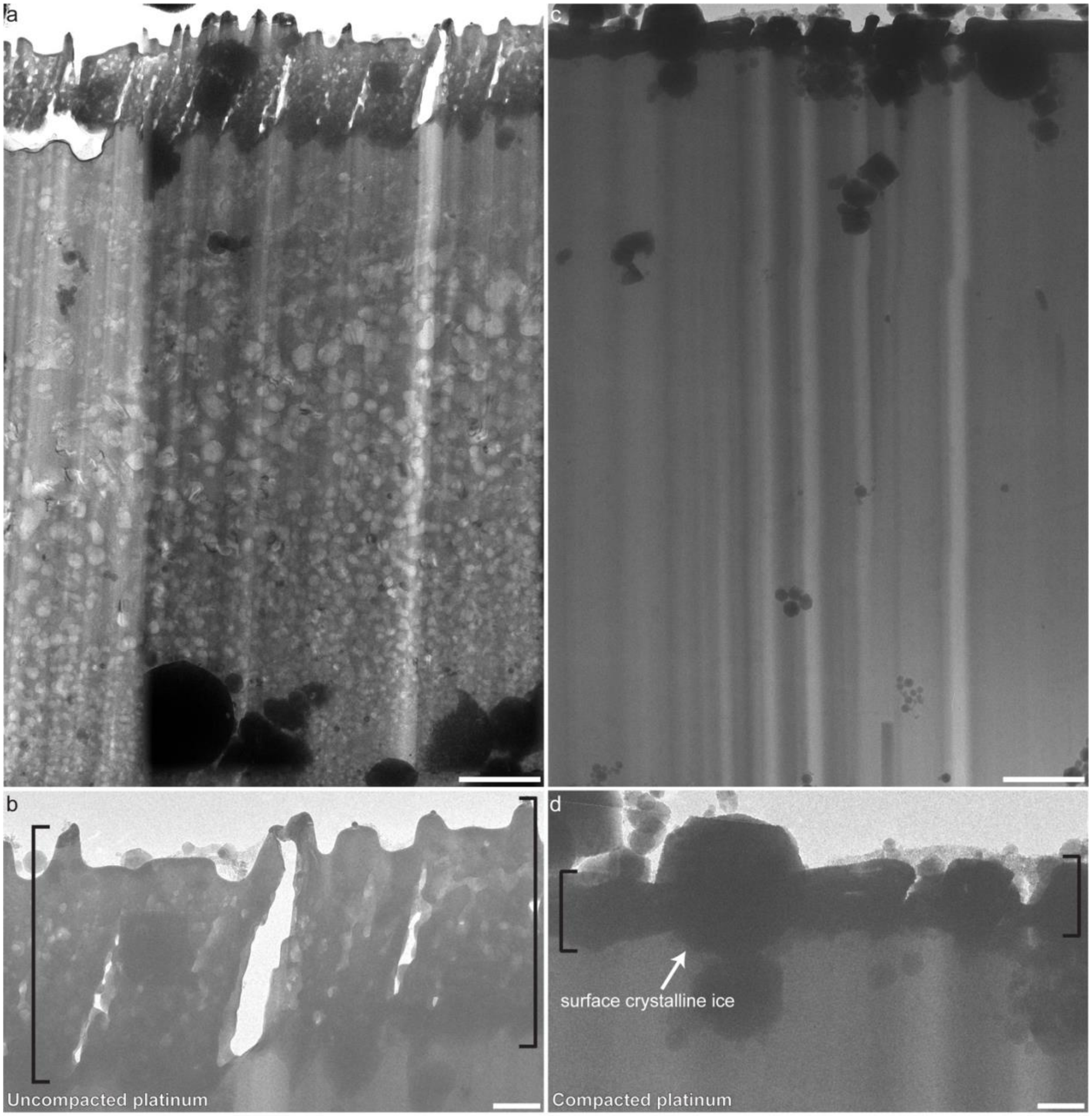


**Supplementary Fig. 3: Intermittent curing effectively compacts organometallic platinum deposition for adequate lamella protection. a** 2D projection image of lamella at x4,800 where platinum was cured only after all platinum was deposited showing clear damage from the FIB from porous platinum architecture (**b**, brackets indicate location of platinum coating). **c** 2D projection image of lamella at x4,800 where platinum was cured intermittently during deposition showing compact platinum architecture (**d**, brackets indicate location of platinum coating). The

compact platinum architecture was observed in 9 distinct lamellae. Scale bars, 2 µm **a** and **c**, 500 nm **b** and **d**.

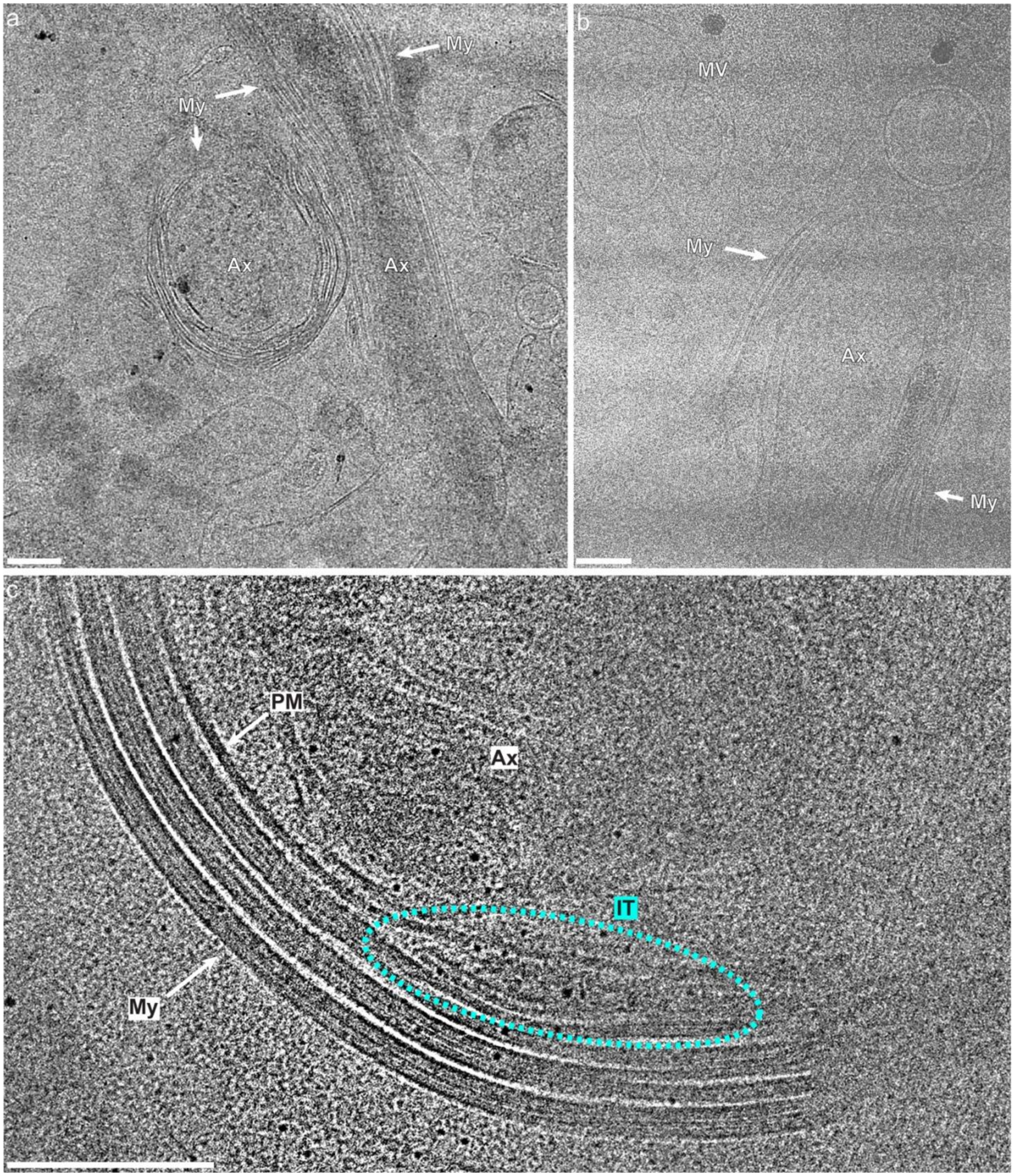


**Supplementary Fig. 4: 2D projection images showing diverse cellular features within lamellae. a-c** Images taken at x19,500 showing diverse cellular features including myelinated (My) axons (Ax), multi-layered vesicles (MV), and the inner tongue (IT) of an oligodendrocyte –

a cytoplasmic expansion near the plasma membrane (PM) of an axon that is thought to be important for continued myelin generation. Scale bars, 200 nm.

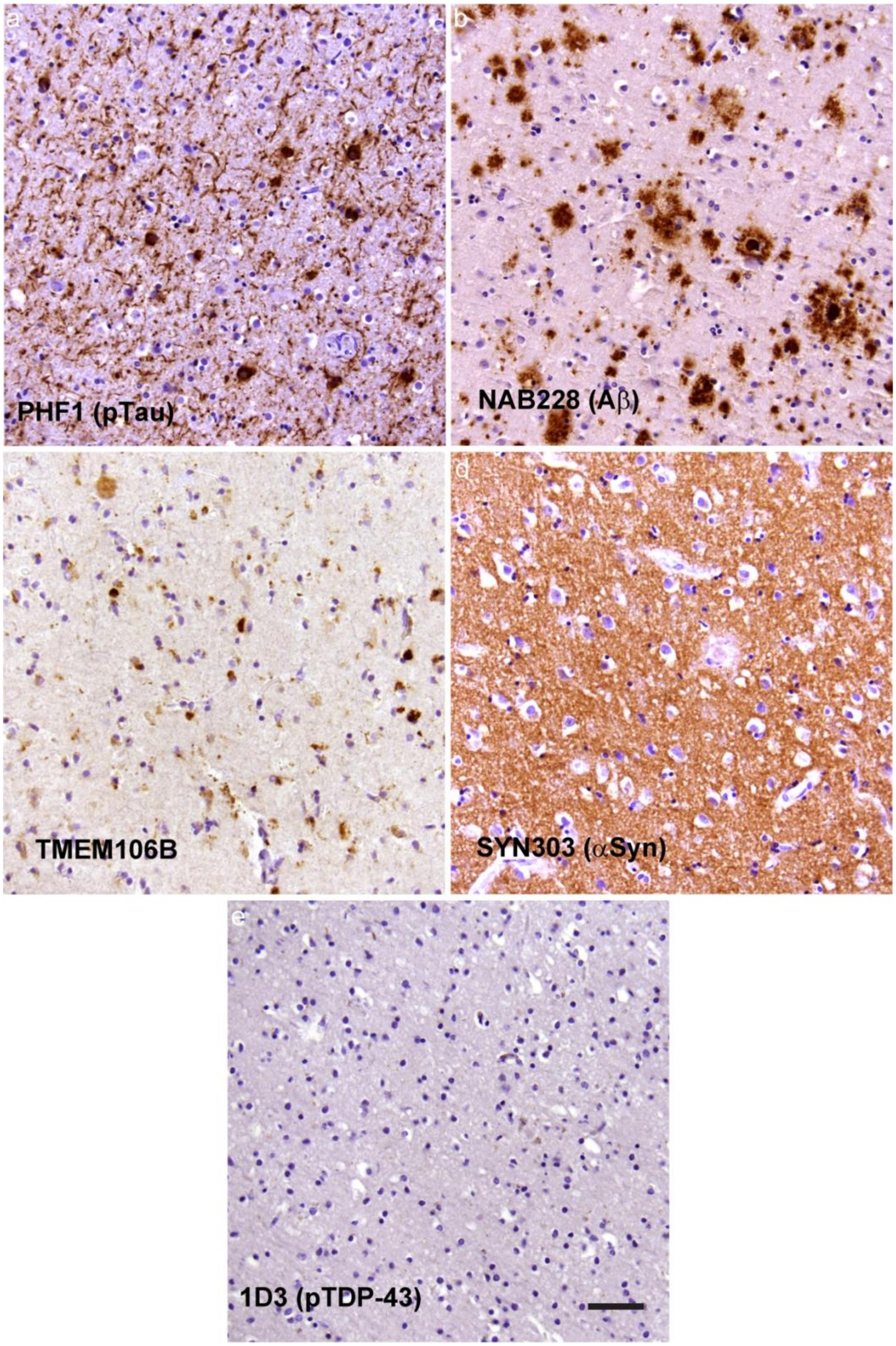


**Supplementary Fig. 5: Immunohistochemical probing for common neurodegenerative**

**disease aggregates in tissue adjacent to the region imaged via cryo-ET.** Positive detection of phospho-tau (pTau) (**a**), amyloid-β (Aβ) (**b**), and TMEM106B (**c**) aggregates and no detection of α-synuclein (αSyn) (**d**) or phospho-TDP-43 (pTDP-43) (**e**) in tissue sectioned immediately adjacent to tissue imaged via cryo-ET. Scale bar, 50 μm.

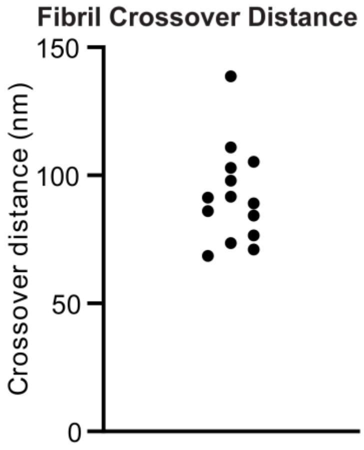


**Supplementary Fig. 6: Crossover distances of fibrils from tomogram in Fig. 4**

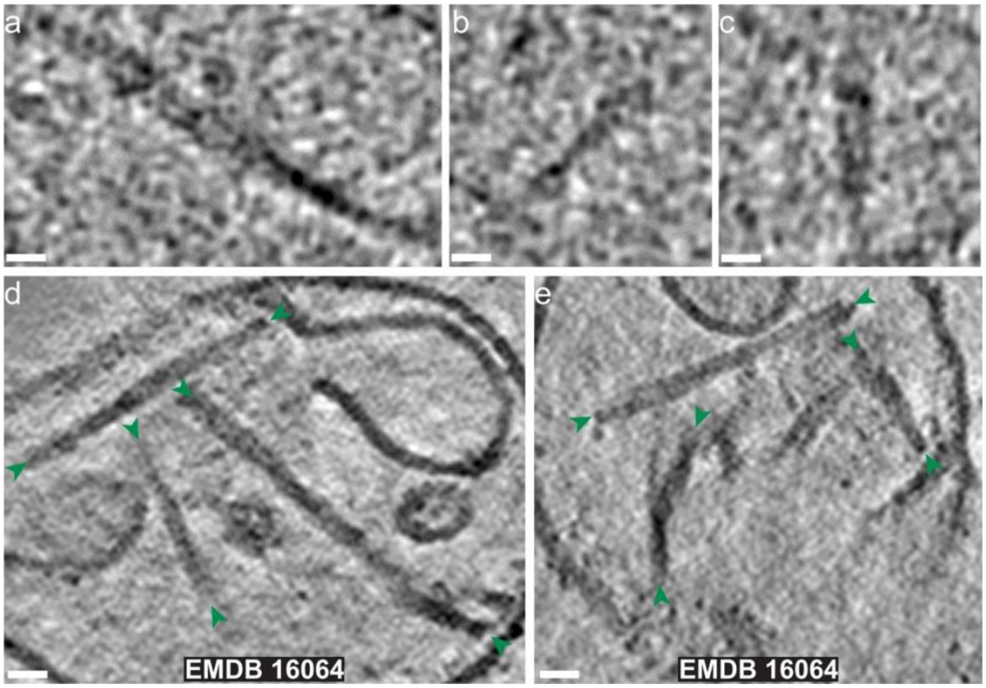


**Supplementary Fig. 7: Potential tau fibrils seen in lamellae generated directly in human brain tissue look similar to potential tau fibrils seen in extracellular vesicles extracted from human brain. a-c** Example potential tau fibrils from tomogram cross-section (the same images used in Fig. 4d-f). **d** and **e** Example cross-sections from tomogram of potential tau (green arrowheads at ends of fibrils) within extracellular vesicles purified from human brain (EMDB:16064)[33]. Scale bars, 20 nm.

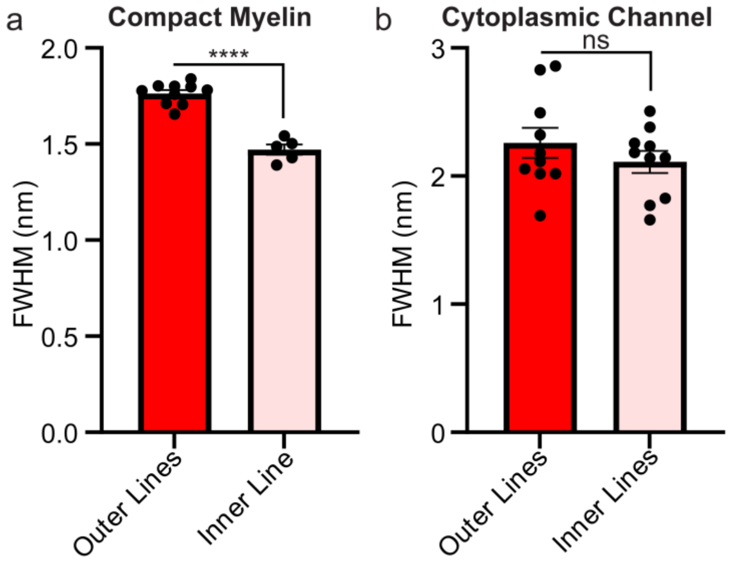


**Supplementary Fig. 8: Full width half maximum (FWHM) measurements of subtomogram averages of compact myelin and cytoplasmic channel myelin. a** and **b** FWHM from average pixel intensity of five adjacent regions of ten Z slices in subtomogram averages of compact myelin (**a**) and cytoplasmic channel myelin (**b**) (points are individual measurements; bars are mean ± SEM; Student's t-Test: ****p<0.0001, p=0.33 for **b**).

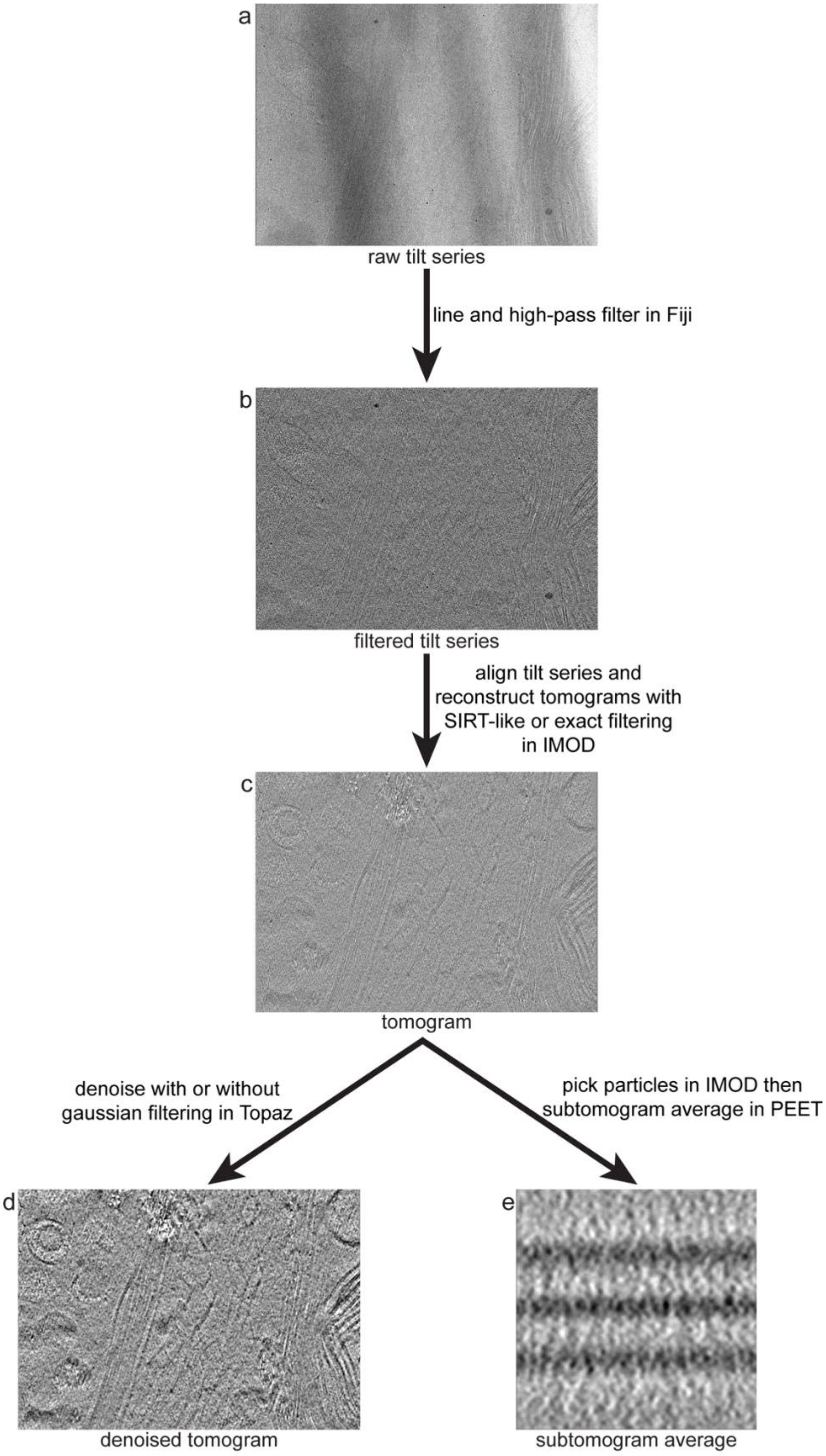

a
raw tilt series
line and high-pass filter in Fiji
b
filtered tilt series
align tilt series and reconstruct tomograms with SIRT-like or exact filtering in IMOD
c
tomogram
denoise with or without gaussian filtering in Topaz
pick particles in IMOD then subtomogram average in PEET
d
denoised tomogram
e
subtomogram average

**Supplementary Figure 9: Cryo-ET data processing workflow.** Raw tilt series (**a**) were line filtered and high-pass filtered in Fiji (**b**) then aligned and reconstructed into tomograms (**c**) in IMOD. After reconstruction, tomograms were denoised in Topaz with or without gaussian filtering for visualization (**d**) or particles were picked in IMOD and subtomogram averaged in PEET (**e**).
