# Supplementary material for: Ultrastructure of human brain tissue vitrified from autopsy revealed by cryo-ET with cryo-plasma FIB milling
Source: Nat Commun. 2024 Mar 26;15:2660. doi: 10.1038/s41467-024-47066-1 (PMC10965902; doi:10.1038/s41467-024-47066-1)
Supplement: Supplementary file 2 — Reporting Summary [file 41467_2024_47066_MOESM2_ESM.pdf]

nature portfolio

Corresponding author(s): Edward B. Lee, Yi-Wei Chang

Last updated by author(s): Feb 26, 2024

Reporting Summary

Nature Portfolio wishes to improve the reproducibility of the work that we publish. This form provides structure for consistency and transparency in reporting. For further information on Nature Portfolio policies, see our Editorial Policies and the Editorial Policy Checklist.

Statistics

For all statistical analyses, confirm that the following items are present in the figure legend, table legend, main text, or Methods section.

n/a	Confirmed	
☐	☒	The exact sample size (n) for each experimental group/condition, given as a discrete number and unit of measurement
☐	☒	A statement on whether measurements were taken from distinct samples or whether the same sample was measured repeatedly
☐	☒	The statistical test(s) used AND whether they are one- or two-sided<br>*Only common tests should be described solely by name; describe more complex techniques in the Methods section.*
☒	☐	A description of all covariates tested
☒	☐	A description of any assumptions or corrections, such as tests of normality and adjustment for multiple comparisons
☐	☒	A full description of the statistical parameters including central tendency (e.g. means) or other basic estimates (e.g. regression coefficient) AND variation (e.g. standard deviation) or associated estimates of uncertainty (e.g. confidence intervals)
☐	☒	For null hypothesis testing, the test statistic (e.g. F, t, r) with confidence intervals, effect sizes, degrees of freedom and P value noted<br>*Give P values as exact values whenever suitable.*
☒	☐	For Bayesian analysis, information on the choice of priors and Markov chain Monte Carlo settings
☒	☐	For hierarchical and complex designs, identification of the appropriate level for tests and full reporting of outcomes
☒	☐	Estimates of effect sizes (e.g. Cohen's d, Pearson's r), indicating how they were calculated

Our web collection on statistics for biologists contains articles on many of the points above.

Software and code

Policy information about availability of computer code

Data collection	SerialEM 4.1
Data analysis	Fiji, IMOD 4.11.24, Topaz 0.2.5, PEET 1.16.0a, ChimeraX 1.6.1

For manuscripts utilizing custom algorithms or software that are central to the research but not yet described in published literature, software must be made available to editors and reviewers. We strongly encourage code deposition in a community repository (e.g. GitHub). See the Nature Portfolio guidelines for submitting code & software for further information.

Data

Policy information about availability of data

All manuscripts must include a data availability statement. This statement should provide the following information, where applicable:

- Accession codes, unique identifiers, or web links for publicly available datasets
- A description of any restrictions on data availability
- For clinical datasets or third party data, please ensure that the statement adheres to our policy

The tomograms shown in this study have been deposited in the Electron Microscopy Data Bank (EMDB) under accession codes: EMD-43334 (Fig. 2d) (https://www.ebi.ac.uk/pdbe/entry/emdb/EMD-43334), EMD-43330 (Fig. 3) (https://www.ebi.ac.uk/pdbe/entry/emdb/EMD-43330), and EMD-43331 (https://www.ebi.ac.uk/pdbe/entry/emdb/EMD-43331) (Fig. 4). The raw tilt series for the tomograms shown in this study have been deposited in the Electron Microscopy Public Image Archive (EMPIAR) under accession code EMPIAR-11920 (https://www.ebi.ac.uk/empiar/EMPIAR-11920).

Research involving human participants, their data, or biological material

Policy information about studies with human participants or human data. See also policy information about sex, gender (identity/presentation), and sexual orientation and race, ethnicity and racism.

Reporting on sex and gender	The example case shown is from a self-reported man. Analysis of sex and gender is not relevant to this study as it is an exploratory technique and does not analyze specific conditions of a disease or physiology.
Reporting on race, ethnicity, or other socially relevant groupings	n/a
Population characteristics	The example case shown is from an 84 year-old man with a 13 year history of behavioral variant frontotemporal dementia. Neuropathologic examination revealed a high level of Alzheimer's disease neuropathologic change (A3, B3, C3), Braak VI, moderate cerebral amyloid angiopathy, and severe arteriosclerosis. Severe tau and amyloid burden were seen in the middle frontal cortex. No TDP-43 or alpha-synuclein were seen.
Recruitment	Participants were recruited to the CNDR brain bank at the University of Pennsylvania through clinical cohorts associated with the Penn Alzheimer's Disease Research Center, Frontotemporal Dementia Center or the Movement Disorder and Parkinson's Disease Center with legal informed consent obtained from the next of kin.
Ethics oversight	Autopsy studies are legally not considered to be human subjects research and so the University of Pennsylvania Institutional Review Board does not provide formal oversight for these studies.

Note that full information on the approval of the study protocol must also be provided in the manuscript.

Field-specific reporting

Please select the one below that is the best fit for your research. If you are not sure, read the appropriate sections before making your selection.

[x] Life sciences [ ] Behavioural & social sciences [ ] Ecological, evolutionary & environmental sciences

For a reference copy of the document with all sections, see nature.com/documents/nr-reporting-summary-flat.pdf

Life sciences study design

All studies must disclose on these points even when the disclosure is negative.

Sample size	No sample size calculation was performed. These are exploratory studies and sample sizes were based on tissue availability at time of autopsy. These samples are sufficient for exploratory methods in technique development as they allowed for determination that the technique can be performed on more than one sample. Additionally, based on tissue availability in over a relatively short period we are limited by the number of individuals that come to autopsy.
Data exclusions	No data was excluded from analyses
Replication	We performed the described protocol on multiple cases testing for vitreous ice as described in all cases. We saw no evidence of crystalline ice within any lamellae and were able to generate lamellae in multiple cases.
Randomization	This is not relevant to our study of exploratory use of FIB-SEM with human tissue as the analyses are not comparing groups and the number of cases does not allow for randomization.
Blinding	This is not relevant to our study of exploratory use of FIB-SEM with human tissue as we are not analyzing differences between different disease or condition types.

Reporting for specific materials, systems and methods

We require information from authors about some types of materials, experimental systems and methods used in many studies. Here, indicate whether each material, system or method listed is relevant to your study. If you are not sure if a list item applies to your research, read the appropriate section before selecting a response.

Materials & experimental systems

n/a	Involved in the study
☐	☒ Antibodies
☒	☐ Eukaryotic cell lines
☒	☐ Palaeontology and archaeology
☒	☐ Animals and other organisms
☒	☐ Clinical data
☒	☐ Dual use research of concern
☒	☐ Plants

Methods

n/a	Involved in the study
☒	☐ ChIP-seq
☒	☐ Flow cytometry
☒	☐ MRI-based neuroimaging

Antibodies

Antibodies used

PHF1 (1:2000) provided by Peter Davies, 1D3 (1:300) provided by Elisabeth Kremmer and Manuela Neumann, TMEM239 (1:500) provided by Michel Goedert, NAB228 (1:30000) provided by Center for Neurodegenerative Disease Research at the University of Pennsylvania, SYN303 (1:10000) provided by Center for Neurodegenerative Disease Research at the University of Pennsylvania

Validation

All antibodies are routinely validated against pathologic human tissue. Validation of antibodies shown in Rye et al. Neuroscience 1993 for PHF1, Neumann et al. Acta Neuropathol. 2009 for 1D3, Schweighauser et al. Nature 2022 for TMEM239, Lee et al. J. Cel Biol. 2005 for NAB228, and Neumann et al. J. Clin. Invest. 2002 for SYN303.

Plants

Seed stocks

Report on the source of all seed stocks or other plant material used. If applicable, state the seed stock centre and catalogue number. If plant specimens were collected from the field, describe the collection location, date and sampling procedures.

Novel plant genotypes

Describe the methods by which all novel plant genotypes were produced. This includes those generated by transgenic approaches, gene editing, chemical/radiation-based mutagenesis and hybridization. For transgenic lines, describe the transformation method, the number of independent lines analyzed and the generation upon which experiments were performed. For gene-edited lines, describe the editor used, the endogenous sequence targeted for editing, the targeting guide RNA sequence (if applicable) and how the editor was applied.

Authentication

Describe any authentication procedures for each seed stock used or novel genotype generated. Describe any experiments used to assess the effect of a mutation and, where applicable, how potential secondary effects (e.g. second site T-DNA insertions, mosiacism, off-target gene editing) were examined.
