# Supplementary material for: Ultrastructure of human brain tissue vitrified from autopsy revealed by cryo-ET with cryo-plasma FIB milling
Source: Nat Commun. 2024 Mar 26;15:2660. doi: 10.1038/s41467-024-47066-1 (PMC10965902; doi:10.1038/s41467-024-47066-1)
Supplement: Supplementary file 8 — Description of Additional Supplementary Files [file 41467_2024_47066_MOESM8_ESM.pdf]

Title: Supplementary Movie 1:
Description: A movie panning through slices of the tomogram shown in Fig. 2d-e with an autophagic vesicle and granular vesicles labeled. Scale bar is 100 nm.

Title: Supplementary Movie 2:
Description: A movie panning through the slices of the tomogram shown in Fig. 3 with examples of granular vesicles, autophagic vesicles, axons (myelinated and unmyelinated), and phagophore-like structures (Ph) labeled. Scale bar is 100 nm.

Title: Supplementary Movie 3:
Description: A movie panning through slices of the tomogram shown in Fig. 4 and Fig. 5 showing a myelinated axon. Scale bar is 100 nm.

Title: Supplementary Movie 4:
Description: A movie of the same tomogram from Supplementary Movie 3, Fig. 4, and Fig. 5a,c with three-dimensional denoising and gaussian filtering to better visualize potential tau fibrils running parallel to the myelinated axon. Examples of potential tau fibrils within the tomogram are labeled. Scale bar is 100 nm.

Title: Supplementary Movie 5:
Description: A movie panning through slices of the tomogram shown in Fig. 4 and Fig. 5 showing a myelinated axon with segmentation of the myelin. Red and blue alternate layers of myelin with darker shades indicating the extracellular surface and the lighter shades representing the intracellular surface of an oligodendrocyte. The layer of myelin closest to the axonal plasma membrane (PM; yellow) has a cytoplasmic channel – a gap between intracellular surfaces of the oligodendrocyte. Scale bar is 100 nm
